# A Proof-of-Concept Electrochemical Skin Sensor for Simultaneous Measurement of Glial Fibrillary Acidic Protein (GFAP) and Interleukin-6 (IL-6) for Management of Traumatic Brain Injuries

**DOI:** 10.3390/bios12121095

**Published:** 2022-11-30

**Authors:** Sarah Shahub, Kai-Chun Lin, Sriram Muthukumar, Shalini Prasad

**Affiliations:** 1 Department of Bioengineering, University of Texas at Dallas, Richardson, TX 75080, USA; 2 EnLiSense LLC, Allen, TX 75013, USA

**Keywords:** traumatic brain injury, SWEATSENSER, Interleukin-6, flexible sweat sensors, passive sweat

## Abstract

This work demonstrates the use of a noninvasive, sweat-based dual biomarker electrochemical sensor for continuous, prognostic monitoring of a Traumatic Brain Injury (TBI) with the aim of enhancing patient outcomes and reducing the time to treatment after injury. A multiplexed SWEATSENSER was used for noninvasive continuous monitoring of glial fibrillary acidic protein (GFAP) and Interleukin-6 (IL-6) in a human sweat analog and in human sweat. Electrochemical impedance spectroscopy (EIS) and chronoamperometry (CA) were used to measure the sensor response. The assay chemistry was characterized using Fourier Transform Infrared Spectroscopy (FTIR). The SWEATSENSER was able to detect GFAP and IL-6 in sweat over a dynamic range of 3 log orders for GFAP and 2 log orders for IL-6. The limit of detection (LOD) for GFAP detection in the sweat analog was estimated to be 14 pg/mL using EIS and the LOD for IL-6 was estimated to be 10 pg/mL using EIS. An interference study was performed where the specific signal was significantly higher than the non-specific signal. Finally, the SWEATSENSER was able to distinguish between GFAP and IL-6 in simulated conditions of a TBI in human sweat. This work demonstrates the first proof-of-feasibility of a multiplexed TBI marker combined with cytokine and inflammatory marker detection in passively expressed sweat in a wearable form-factor that can be utilized toward better management of TBIs. This is the first step toward demonstrating a noninvasive enabling technology that can enable baseline tracking of an inflammatory response.

## 1. Introduction

A traumatic brain injury (TBI) is defined as a neurotrauma caused by a mechanical force that is applied to the head. In the United States, there are approximately 1.7–2.0 million incidents of TBI annually. In addition, the centers for disease control reported that approximately 5.3 million Americans live with the effects of a TBI. About half of the Americans who experience a TBI each year incur at least some form of short-term disability. Currently, a TBI can also be classified by its Glasgow Coma Scale (GCS) score: severe (GCS 3–8), moderate (GCS 9–12), and mild (GCS 13–15), as well as by cranial computer tomography (CT) abnormality (CT positive vs. CT negative) [1]. Mild TBIs (mTBI, CT negative) account for over 85% of all cases of TBIs. In the U.S., TBIs account for 1.3% of all emergency department visits [2]. The direct medical costs for treatment of TBIs in the U.S. have been estimated to be $4 billion annually.

The GCS method depends on a scale which is used by physicians to evaluate suspected TBI patients based on their eye, motor, and verbal responses, for which a numerical score is assigned to indicate the severity of a TBI [3]. This method of diagnosing a TBI is widely used because it can be assessed immediately and noninvasively. However, this method presents a risk of human error in diagnosis, especially in the case of a mild TBI, where the patient may not express many of the symptoms after their initial injury [4]. The CT scan method is more accurate than the Glasgow Coma Scale in identifying a TBI. However, CT scanning cannot be performed immediately after injury, as it requires both the use of specialized equipment and a qualified specialist to interpret CT scans. This method therefore presents a significant delay in obtaining treatment for a TBI patient [5].

There is a consensus in this field that there is an unmet medical need for a rapid, simple, biofluid-based diagnostic test for the management of TBI patients. Recently, there have been many studies demonstrating that biofluid-based TBI biomarker tests have the potential to assess the extent of TBI severity and determine a patient prognosis even at times when correlation with other neurological measures (neuroimaging) may not always be informative, such as in the case of a mild TBI.

Glial fibrillary acidic protein (GFAP) is a type III intermediate filament protein that provides structural support to the Central Nervous System (CNS) [6]. The presence of GFAP in serum has been linked to TBIs in clinical studies of serum post-injury. Serum of TBI patients showed elevated levels of GFAP up to 30 days after injury, with GFAP levels peaking within the first hour after injury [7].

Glial fibrillary acidic protein (GFAP) is emerging as the most robust TBI biomarker (GFAP biomarker levels are elevated within 3 to 34 h in cerebrospinal fluid and serum/plasma following a severe TBI [8], and in serum and plasma samples after a moderate to mild TBI [9]). GFAP as a biomarker, in the form of either the GFAP intact protein (50 kDa) or as the breakdown products (GFAP-BDPs; 44–38 kDa), is predominantly released from injured brain tissue into biofluid such as cerebrospinal fluid and serum/plasma shortly following a TBI [10]. GFAP has been shown to be expressed in human keratocytes and in the epidermis.

Interleukin-6 (IL-6) is an inflammatory cytokine released by immune cells in response to stress, infection, or injury to induce inflammation as a defensive mechanism against further infection or trauma [11]. The release of IL-6 is a secondary physiological response; therefore, IL-6 has a slower peak response time than GFAP (5–8 h) after injury and remains elevated in the blood for a longer period of time [12].

IL-6 (21–28 kDa) is the cytokine found in the largest quantity in circulation in response to inflammatory or traumatic insults, acting in both systemic and local responses [13]. IL-6 acts as a cellular mediator for immunological responses and can be produced through inflammatory events in the central nervous system (CNS) [14]. We have previously demonstrated the presence of IL-6 in human sweat. We have also demonstrated through human clinical studies that the IL-6 expression profiles in sweat correlated with systemic expression in serum [15].

The current standard of GFAP detection is through serum testing, to which end multiple studies have developed serum-based sensors for GFAP for TBI detection. Agostini et al. developed three antibody-based Lab-on-Chip sensors to measure GFAP in serum; their sensing platforms were each designed to resolve a different issue inherent to the sensor, i.e., antifouling, antibody orientation, and probe surface density [16]. Khetani et al. designed a GFAP sensor utilizing polyethylenimine (PEI)-coated graphene screen-printed electrodes for the detection of TBIs, from which the sensor response was characterized for GFAP spiked in different mediums: phosphate buffer saline, artificial cerebrospinal fluid, and human serum [17]. Another study focused on quantifying the levels of GFAP in serum for the purpose of detecting TBIs and neurodegeneration. Wang et al. used screen-printed carbon electrodes in their electrochemical sensor design, the performance of which was evaluated in human blood serum [18].

Though serum testing has been widely used for GFAP and IL-6 detection, the constraints of serum testing present challenges toward continuously monitoring TBI progression post-injury. Serum testing is highly invasive and requires temperature-controlled storage from the time of collection to testing and the maintenance of sterile conditions for the collected samples. These constraints contribute to making serum testing a highly cumbersome and time-consuming process. Time-based tracking of TBIs is not possible through serum testing, largely because of the long response time involved in serum collection and testing.

Considering the challenges that prevent serum testing from being a viable method for continuous tracking of TBIs post-injury, there exists an opportunity for sweat as a biofluid for noninvasive, continuous monitoring of biomarkers. This work focuses on using passively expressed sweat to simultaneously detect GFAP and IL-6 for continuous tracking of TBI progression after injury.

Table 1 summarizes the expression profile of GFAP and IL-6 in healthy and TBI subjects in human serum/plasma. The expression profile in human sweat for IL-6 is well understood, whereas it is poorly understood for GFAP. Hence, for this study we have based the sensor design and demonstration of proof of feasibility based on the current understanding in clinical science of the expression profiles of these molecules.

Human sweat is a noninvasively collectable biofluid that hosts a broad range of biomarkers. In the past decade, there has been significant interest in the analysis and investigation of sweat components, leading to the current importance of sweat as a potential diagnostic biofluid. The use of sweat-based wearable sensors can enable a new technological horizon, namely continuous noninvasive sensing devices, by offering insight into the dynamics of the human body at the molecular level. Recent studies of wearable devices that provide an interface directly with skin and that can be worn in various locations on the body have shown that sweat is a suitable biofluid for many potential clinical applications, such as health management, sports performance, and disease diagnosis [23]. The molecular components in sweat can provide a wide range of information about the human physiological state by measuring a broad range of biochemical compounds, ranging from metabolites (e.g., glucose, urea, lactate, cortisol, and ethanol) and electrolytes (e.g., potassium, sodium, chloride, ammonium, zinc, and copper) to large molecules (e.g., DNA, peptides, proteins, and cytokines).

Hence, in this study we have explored for the very first time an electrochemical sensor to measure and report GFAP and IL-6 in human sweat toward the proof-of-feasibility demonstration of a sensor platform with the capability for prognostic tracking of TBIs using biomarkers. Analysis of sweat biomarkers has primarily been achieved by a method of electrochemical sensing using biosensors. A biosensor is an analytical device used to provide real-time data (such as concentration) of one or more chemical constituents (analytes) in a sample [24]. An electrochemical biosensor is an analytical device that transduces biochemical events such as antigen–antibody interactions to electrical signals (e.g., current, voltage, impedance, etc.) [25]. Recently, various electrochemistry-driven biosensing methods have been introduced for simple and miniaturized analytical devices for skin-based sensor analysis [26].

Although sweat can offer a large amount of physiological information for disease detection, drawbacks in previous studies using biosensors include poor sweat collection methods, separate collection and analysis stages, and an inability to monitor multiple analytes simultaneously [27]. Hence, in this work we address these challenges by demonstrating for the very first time the measurement of GFAP and IL-6 in a simultaneous manner from passive eccrine sweat using an electrochemical sensor platform. We have demonstrated the proof of feasibility in exogenously induced low and high expression profiles for distinguishing the conditions of mild, moderate, and elevated TBIs.

## 2. Materials and Methods

### 2.1. Sensor Platform

**Figure 1:**
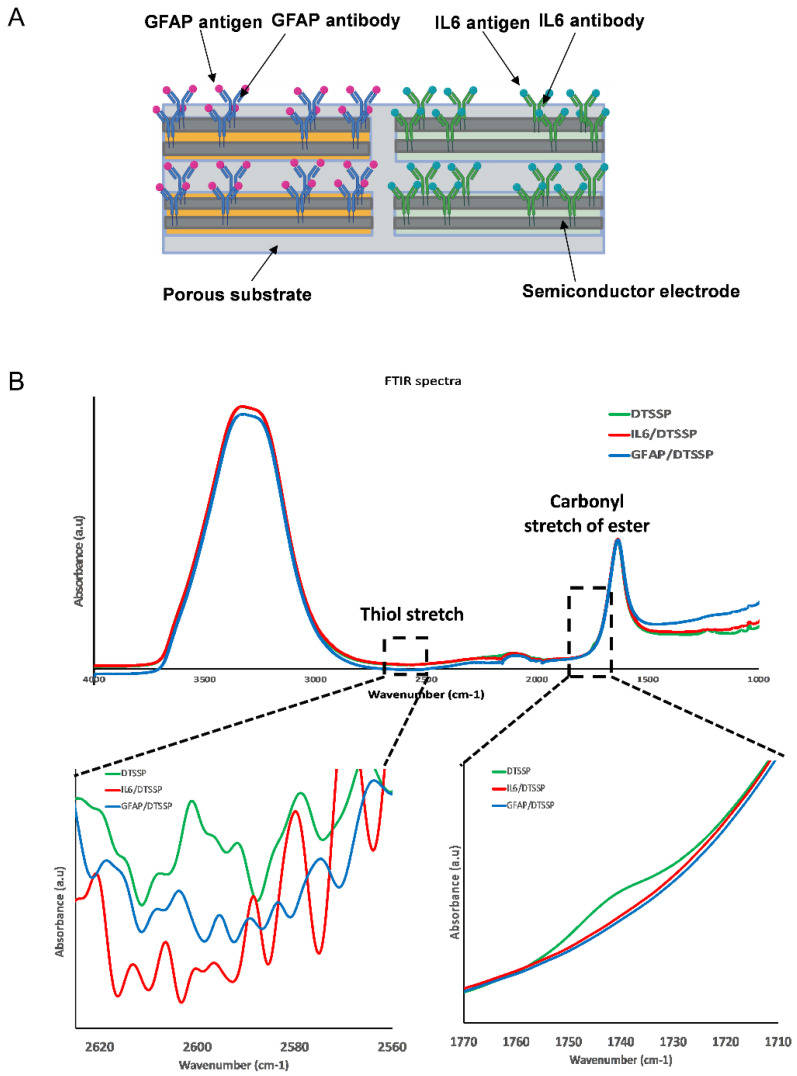
(**A**) Sensor design and surface chemistry. (**B**) FTIR of DTSSP, GFAP antibody, and IL-6 antibody on ZnO.

The sensor comprises a sweat-sensing strip with four pairs, i.e., eight electrodes functionalized for specific target biomarkers that can be connected to a portable potentiostat that transduces the impedance from the sensor to output a calibrated concentration of the measured biomarker levels in sweat. The process of sensor fabrication has been described in detail previously by our group [28]. Briefly, a 2-electrode system was fabricated through the screen-printing technique, which enables the transduction of the affinity-based interaction between the target biomarker and capture probe antibody to produce a measurable electrochemical signal. Figure 1A demonstrates the schematic of the sensor device.

The wearable device reader utilizes the Analog Devices ADuCM350 system-on-chip (SoC) system as part of a four-part system. The SoC implements the impedance measurement, while the Nordic nRF8001 Bluetooth chipset establishes the Bluetooth connection. Temperature and relative humidity measurements are taken by the built-in Texas Instruments HDC1080 sensor, and the Texas Instruments BG24040 is utilized as the Lithium-ion battery power system. The Analog Front End of the device is composed of a wave generator, a switch matrix, and a 2048-point discrete Fourier transform-based impedance analyzer. Two of the analog pins are designated as the generator and sink, which connect to one of the sensors through the switch matrix. A 13-millisecond discrete Fourier transform cycle precedes the AFE switching to the next sensor channel, which continues until all four sensors have been measured for impedance, in order to produce multiple replicates of the same measurement [29].

The substrate used in the fabrication of the sensor is a porous polyamide membrane that allows for confinement of the target biomarkers. The porous membrane reduces diffusion time of the molecules to the sensor surface and provides a high surface to volume ratio, thus enhancing the sensor capability. The porous membrane also reduces interference of non-specific molecules by providing size-based exclusion of larger molecules, thus increasing sensor selectivity [30].

### 2.2. Reagents and Instrumentation

3,3′-dithiobis(sulfosuccinimidyl)propionate (DTSSP) cross-linker and 10 mM phosphate buffered saline (PBS) were procured from Thermo Fisher (Waltham, MA, USA). Monoclonal IL-6 GFAP antibodies, GFAP protein, and IL-6 proteins were purchased from Abcam (Waltham, MA, USA). Polyamide substrates were ordered from GE Healthcare Life Sciences (Piscataway, NJ, USA) with 0.2 μm pore size, product number 2107356-001. Single donor human sweat was purchased from Lee Biosolutions Inc. (St. Louis, MO, USA) and spiked with GFAP and IL-6 in order to simulate the sweat of a TBI patient immediately after injury.

The description of the sweat sensor has been provided in detail previously [15,28,31]. Briefly, the sensor device had a sensor strip and a wearable reader. The sensor strip was immobilized with GFAP and IL-6 antibody and connected to the reader using a connector. This entire wearable was placed on the hand such that the sensor strip interfaces firmly with the skin to absorb low volumes of passive sweat. The GFAP and IL-6 from diffused sweat were captured by their respective antibodies and a corresponding electrochemical signal was measured. The electrochemical signal was measured using impedance spectroscopy transduction mechanism. A sinusoidal input voltage (10 mV) was applied and the resulting impedance owing to the binding of the target molecule to the capture probe antibody was recorded over a frequency range of 10,000–1 Hz [32,33]. A calibration curve was developed by measuring the impedance response for varying concentrations of the target analytes at 180 Hz.

### 2.3. Immunoassay Functionalization

The capture probe antibody was covalently linked to the sensor surface via a thiol cross-linking mechanism. For this, 10 mM DTSSP was mixed in separate solutions with 1 μg/mL GFAP antibody and 10 μg/mL IL-6 antibody. The mixed solution was incubated at room temperature for 30 min and immediately dispensed on the sensing electrode and incubated overnight at 4C before use. During incubation, 30 µL of the antibody in liquid form was dispensed onto each sensor, which equates to 7.5 µL of antibody dispensed per sensing unit (four per sensor).

The type of antibodies used in this study were monoclonal GFAP and IL-6 antibodies purchased from Abcam (Waltham, MA, USA). Monoclonal antibodies are lab-synthesized antibodies from identical cells that originate from a single parent cell. They are made specifically to target one antigen and can achieve a high degree of specificity due to their single origin cell. Monoclonal antibodies for GFAP and IL-6 were chosen due to their high specificity for their respective target antigens.

FTIR was conducted using the Nicolet iS-50 (ThermoFisher Scientific Inc., Waltham, MA, USA) with samples of the linker molecule and GFAP and IL-6 antibodies prepared according to the protocol outlined in Section 3.1. The samples were deposited on a ZnO-coated glass slide, and the measurements were recorded in attenuated total reflectance mode. The number of spectral scans recorded was 256 with a resolution of 4 cm^−1^ over a wavelength range of 4000 to 1000 cm^−1^ [15].

### 2.4. Evaluation of Sweat Sensor Performance

The sensor performance metrics were established to ensure the device was able to detect GFAP and IL-6 reliably. A calibration was first developed using in vitro experiments. Varying concentrations of GFAP from 7.81 to2664 pg/mL and IL-6 from 0.78 to 766.4 pg/mL were dispensed on the sensor and the impedance response was measured. The obtained impedance response was used to build the calibration curve using a 4-parameter fit. Once the calibration curve was established, performance metrics through spike and recovery, reproducibility, and specificity experiments were evaluated. For the spike and recovery experiment, various concentrations from low to high levels for each target biomarker was spiked on the sensor and the measured impedance response was used to compute the estimated concentration using the developed calibration curve. Reproducibility of the sensor was evaluated by taking triplicate measurements of the sensor response to synthetic sweat spiked with low and elevated concentrations of GFAP (39 pg/mL and 2664 pg/mL) and IL-6 (16 pg/mL and 766 pg/mL) on separate sensing units. Specificity of the sensor to GFAP and IL-6 was evaluated by spiking low and high concentrations of the nonspecific molecule IL-6 (6.2 pg/mL and 166.7 pg/mL) and GFAP (10 pg/mL and 810 pg/mL) to measure the non-specific sensor response to either marker. Benchtop experiments were performed at room temperature.

### 2.5. Statistical Analyses

All statistical tests were performed in GraphPad Prism. Unpaired t-test analysis was used to find the significance between the sensor response to low and high concentrations of GFAP and IL-6. One-way ANOVA analysis was performed to determine the significance between the sensor response to sweat analogs spiked with different concentrations of GFAP and IL-6. The data is represented as mean ± S.E. A minimum of n ≥ 3 measurements were performed for the calibration and spike and recovery experiments.

## 3. Results and Discussion

### 3.1. Sensor Design

The sensor platform has been described in detail elsewhere [15,28,31]. Briefly, the sensor platform was designed with four zinc oxide (ZnO)-coated electrode pairs on a porous substrate. The four-sensor layout consists of four pairs with a working electrode and a reference electrode in each pair. The working electrode receives the sinusoidal input signal, while the reference electrode acts as the reference against which the signal change is measured. Each electrode measures 0.4 cm in length and 0.2 cm in width and the entire sensing area covered by the electrodes and substrate measures 1 cm in length and 0.4 cm in width. Two electrode pairs were functionalized with the GFAP antibody, and two electrode pairs were functionalized with the IL-6 antibody. The porous substrate was designed to provide a medium for collection of passive sweat from the skin surface, which diffuses to the electrodes and is collected in the volume range of 1–5 µL. Figure 1A depicts the GFAP and IL-6 antibody on the semiconductor electrode surface; the antibodies act as the capture probes for the target analytes, GFAP and IL-6. Sweat is transported from the skin surface to the electrodes via the porous substrate. The GFAP and IL-6 antibodies are bound to the sensor surface via the DTSSP cross-linker.

A ZnO semiconductor was chosen as the electrode material due to its stability at different pHs, according to previously conducted studies to characterize the sensor system [30]. The stability of the sensor was evaluated in samples of synthetic sweat formulated at different pH levels. Repeat measurements of the sensor response were recorded for the samples at different pH levels and found to vary within 7%. The pH of sweat can vary among individuals; therefore, ZnO was chosen as the material for the active sensing element in the design due to its stability over a pH range of 4.0 to 8.0.

ZnO was also chosen as the active sensing element in our electrodes due to its properties as a biocompatible semiconductor material with a wide band gap of 3.37 eV and high isoelectric point (IEP) of ~9.5. The electrical properties of ZnO are also customizable, as ZnO is positively charged at pH 7. ZnO’s higher band gap gives it an advantage in the form of a higher breakdown voltage and the ability to sustain larger electric fields, while the IEP also enhances the adsorption of proteins to the sensor surface due by increasing the electrostatic attraction between the target and sensor surface.

Moreover, the surface terminations in the structure of ZnO can be used to increase sensor specificity. Alternate deposition conditions can be used to achieve an n-type ZnO material that grows in wurtzite crystal formed of alternate stacked layers with zinc and oxygen terminations. This specialized ZnO structure has been previously used for the functionalization of linker molecules such as Dithiobis [Succinimidyl Propionate] (DSP) and (3-Aminopropyl) triethoxysilane (APTES) to enhance the sensitivity to adsorbed molecules [34,35]. Using ZnO as the active sensing material gives the ability to modulate the surface states in order to achieve increased specificity of the sensor [36].

Figure 1B depicts the immobilization of the capture probe via thiol binding of the cross-linker to the sensor surface for both IL-6 and GFAP antibodies, respectively. The top of Figure 1B depicts the whole wavenumber range of FTIR spectrum from 1000 to 4000 cm^−1^. The carbonyl stretch of ester bond peak disappears once the antibody is bonded. The peak (1740 cm^−1^) of cross-linker DTSSP binding to the IL-6 and GFAP antibodies can be seen at the bottom right zoomed-in portion of the figure. The conjugation of the antibody and the DTSSP cleaves the C-O bond of the NHS ester bond, leading to the disappearance of the peak. The S-H stretch can be expected between 2550 and 2620 cm^−1^ and indicates the stretch of the S-H bond binding the sulfur to the ZnO surface. The S-H stretch signal is very weak but still can be seen at 2600 cm^−1^, showing the disappearance of the peak of the IL-6/DTSSP conjugation and the GFAP/DTSSP conjugates, respectively. Following functionalization of the IL-6 and GFAP antibodies on the ZnO surface, the absorbance peak disappears, indicating the immobilization of the capture probe. Therefore, we can hypothesize that the antibody capture probe has bound to the surface.

### 3.2. Electrochemical Sensor Response for GFAP and IL-6

The sensor used in this study was an impedimetric sensor functionalized with both GFAP and IL-6 antibodies for simultaneous sensing of both biomarkers. The method of measurement used was electrochemical impedance spectroscopy (EIS). EIS detection depends on the change in impedance in response to a sinusoidal input signal applied across the working and reference electrodes. The impedance is modulated due to the change in capacitance in response to binding of GFAP and IL-6 to their antibody capture probes, which are bound to the electrode surface via a cross-linker.

In EIS, the application of the input signal results in the formation of a double positive–negative layer of charged molecules bound to the surface, known as an Electric Double Layer (EDL). The EDL behaves as a capacitor, and its inherent capacitance is modulated in response to binding of the target analytes to their respective capture probes. As a result of the amount of the target that binds to the surface receptor depending on both its concentration and affinity for the receptor, the greater the concentration of the target analyte, the greater the signal response will be and vice versa [37].

**Figure 2:**
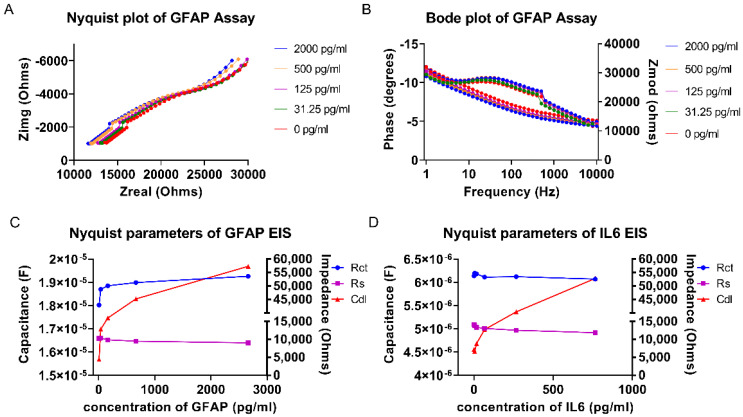
(**A**) Nyquist plot of GFAP EIS assay. (**B**) Bode plot of GFAP EIS assay. (**C**) Nyquist parameters of GFAP EIS assay. (**D**) Nyquist parameters of IL-6 EIS assay.

Figure 2A,B depicts the Nyquist and Bode plots of the sensor response to GFAP spiked in sweat analog in the concentration range of 0 to 2000 pg/mL using EIS at the frequency range of 10,000 to 1 Hz. From the Nyquist plot, a dose dependent shift in the imaginary impedance can be observed from 0 to 2000 pg/mL of GFAP, which indicates an increase in EDL capacitance. This conclusion is supported by the dose dependent shift in the phase, as shown in Figure 2B. From the Bode plots, it can be observed that the maximum capacitance occurs within the frequency range of 100–500 Hz. The phase of the sensor response experiences an increasingly negative shift as the GFAP concentration increases, which corresponds to the increasing capacitance of the EDL. The sensor demonstrated a similar trend in response to IL-6. The double layer capacitance (Cdl), solution resistance (Rs), and charge transfer resistance (Rct) were calculated from an equivalent circuit and plotted for increasing concentrations of GFAP and IL-6, as shown in Figure 2C,D.

Whenever there is binding between the antigen and the immobilized antibody, charge rearrangement gives rise to capacitive modulations in the EDL, which is represented by an increasing Cdl component of the sensor in response to increasing concentrations of GFAP and IL-6, shown in Figure 2C,D. The amount of charge transfer resistance also increases with increasing target concentration because the antigens are protein targets. With increasing protein binding to the capture probe, a resistance to the transfer of charges occurs. This is evident by an increase in the Rct component. A combination of both the Rct and Cdl response highlights the sensitivity of the platform to GFAP and IL-6 concentration. The range for fitting of the Rct component has a lower dynamic range; therefore, a dominant capacitive effect is observed. Rs, the solution resistance, is caused by bulk solution effects, which ideally should have minimal effect on the overall signal. With highly ionic solutions such as human sweat, the sensor platform is required to be robust enough to withstand bulk effects of the buffer. The bulk solution effect can be observed by the trend of the Rs component of the Randle’s circuit fitting result in Figure 2C,D, where the Rs range does not show any increasing trend with increasing GFAP and IL-6 concentration. It can therefore be concluded that the signal is not affected by the bulk effects of the solution and that the binding phenomenon at the EDL layer is the dominant contributor to the sensor response. Thus, the sensor platform is sensitive to increasing GFAP and IL-6 concentrations and has low noise contribution from the background ions in the sample.

### 3.3. Calibrated Dose Response (CDR) Study Using EIS

Non-faradaic electrochemical impedance spectroscopy (EIS) was used to measure the sensor response. A very low sinusoidal input voltage was applied, and the resulting impedance owing to the binding of the target molecule to the capture probe antibody was recorded over a frequency range of 100 to 1000 Hz. A calibration curve was developed by measuring the impedance response for varying concentrations of the target analyte. Chronoamperometry was used to measure the sensor response. Cyclic voltammetry (CV) studies were carried out to observe the maximum diffusion potential of the sensor when exposed to increasing concentrations of GFAP and IL-6. CV was used to measure the current response of the sensor from −0.8 to 0.8 V. The maximum diffusion current was recorded at 0.6 V. For this reason, chronoamperometry studies were carried out with an offset potential of 0.6 V. The output current owing to the diffusion of the target molecule to the capture probe antibody on the sensor surface was recorded 35 s after introduction of the input voltage. A calibration curve was developed by measuring the current response for varying concentrations of the target analyte.

**Figure 3:**
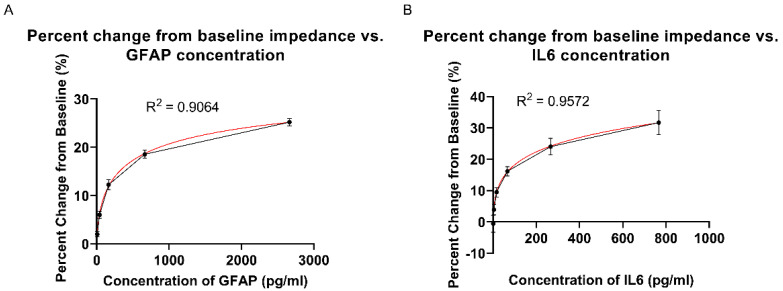
(**A**) GFAP EIS CDR percentage change from impedance baseline vs. GFAP concentration. (**B**) IL-6 EIS CDR percentage change from impedance baseline vs. IL-6 concentration.

Calibration response curves were developed for both GFAP and IL-6 using synthetic sweat spiked in the concentration ranges representing low and elevated concentrations of both markers. GFAP was applied onto the sensor in the range of 7.81–2664 pg/mL, while IL-6 was applied on the sensor in the range of 0.78–766.4 pg/mL. The calibration studies were carried out using two different methods of detection, EIS and chronoamperometry, to compare the response of the sensor across two different methods. Figure 3A,B depicts the sensor response to GFAP and IL-6 using EIS, with the percentage change in the impedance from baseline as a function of spiked concentration, taking as the baseline reading as the sensor response to synthetic sweat. The sensor response is shown to monotonically increase as the spiked concentration increases. The CDR data was fitted to a four-parameter curve, for which the R-squared was greater than 0.9. From this curve, the CDR equation was extracted. The limit of detection (LOD) was estimated by taking the mean concentration of the baseline (blank) dose (synthetic sweat) as measured by the sensor, and the standard deviation of the mean baseline concentration measured, which were used to find the limit of blank (LOB). The calculated LOB value and the standard deviation of the lowest dose was then used to estimate the LOD for both GFAP and IL-6, which were found to be 14 pg/mL for GFAP and 10.22 pg/mL for IL-6, which are consistent with the low physiological ranges of the markers in sweat [38].

### 3.4. Calibrated Dose Response (CDR) Study Using Chronoamperometry

**Figure 4:**
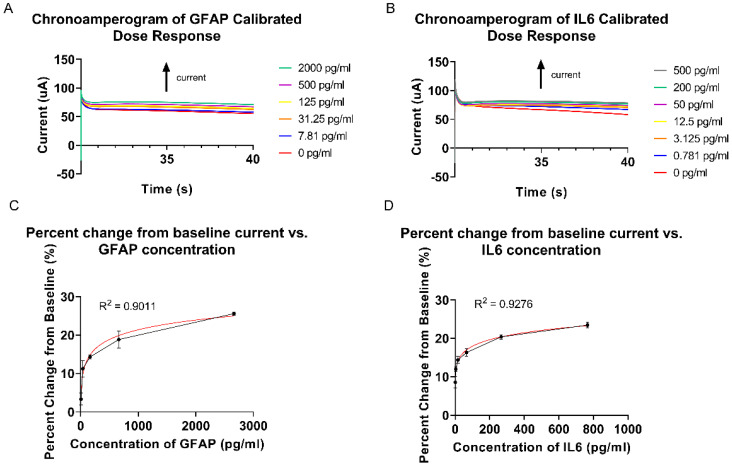
(**A**) Chronoamperogram of GFAP CDR. (**B**) Chronoamperogram of IL-6 CDR. (**C**) GFAP chronoamperometry CDR percentage change from impedance baseline vs. GFAP concentration. (**D**) IL-6 chronoamperometry CDR percentage change from impedance baseline vs. IL-6 concentration.

A CDR study was carried out using chronoamperometry for both GFAP and IL-6 using a sweat analog spiked in the concentration ranges used for the CDR in EIS. Chronoamperometry was used to validate EIS as the electrochemical method of measurement by measuring the diffusion current as the concentration of the target analyte increased—which coincides with increased perturbation of the EDL—as measured through impedance by EIS. Chronoamperometry measures the output current in response to a square wave input signal, which increases as diffusion occurs at the electrode surface. Unlike EIS, the output signal in chronoamperometry depends on the diffusion of the bulk solution molecules and is therefore more susceptible to interference from the bulk solution alongside the target analyte. Figure 4A,B depicts the current over time of the sensor response to GFAP and IL-6 for these spiked concentrations. In order to measure the diffusion current of the system, the current was recorded after 35 s for each concentration, where the current has reached stability. Figure 4C,D shows the sensor response to GFAP and IL-6 using chronoamperometry as the percentage change in the current from baseline as a function of spiked concentration, taking the baseline reading as the sensor response to synthetic sweat, for which the sensor response is shown to monotonically increase as the spiked concentration increases. The CDR data was fitted to a four-parameter curve, for which the R-squared was greater than 0.9. From this curve, the CDR equation was extracted. The CDR obtained from chronoamperometry is comparable to the CDR obtained from EIS, which validates using EIS for measuring the change in EDL in response to binding, as chronoamperometry measures the same change in EDL via the diffusion current.

### 3.5. Spike and Recovery Study in Sweat Analogs

**Figure 5:**
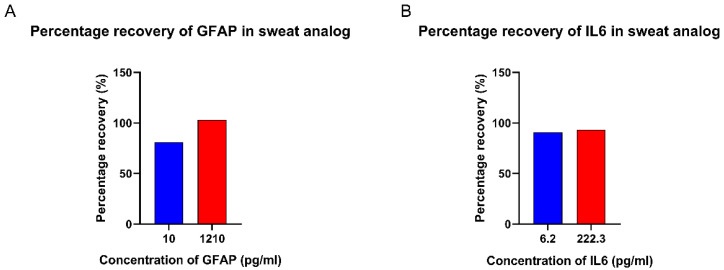
(**A**) Percentage recovery of GFAP in sweat analogs. (**B**) Percentage recovery of IL-6 in sweat analogs.

The sensor performance was validated by spiking sweat analogs, the composition of which is described in work by Mathew et al. [39] at pH 6 with GFAP and IL-6 in low and elevated concentrations within the concentration ranges used to develop the calibration curves in Section 3.3. Using the CDR equations extracted from the EIS data, the spiked concentrations were estimated for GFAP and IL-6 from the sensor response. Figure 5 depicts the recovered concentrations of GFAP and IL-6 estimated by the sensor as a percentage of the actual spiked concentration used. The percentage recovery ranged from 80% to 103% in the case of GFAP, and from 90% to 93% in the case of IL-6, which lies within a 20% standard deviation from 100% recovery. This demonstrates that the sensor is able measure concentrations of GFAP and IL-6 within the lowest to highest concentration ranges of both markers.

### 3.6. Cross-Reactivity Study

**Figure 6:**
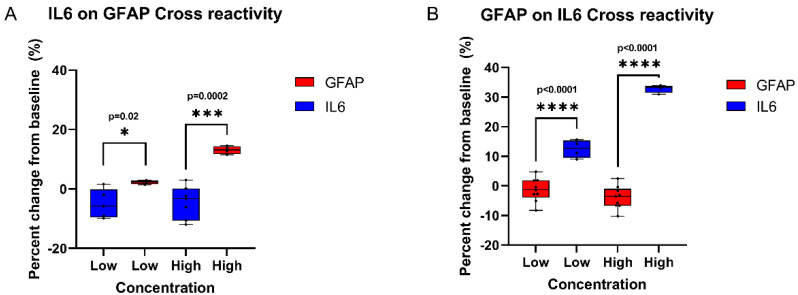
(**A**) Percentage change from baseline of non-specific and specific response of IL-6 on GFAP. (**B**) Percentage change from baseline of non-specific and specific response of GFAP on IL-6.

A cross-reactivity study was carried out using sweat analogs spiked with low and elevated concentrations of GFAP and IL-6. The purpose of this study was to determine whether for the two markers the non-specific marker will cause a significant signal output, which would interfere with the signal output due to specific binding. For minimal cross-reactivity, the non-specific response of the sensor was expected to be significantly lower than the specific signal at both low and elevated concentrations of both markers. The non-specific and specific responses of the sensor to both markers were recorded and compared for cross-reactivity. Figure 6 depicts the percentage change from baseline impedance for GFAP on the area of the sensor functionalized for IL-6, taken as the non-specific response, alongside the sensor response to IL-6 at the same concentration, taken as the specific response. Similarly, the percentage change from baseline impedance for IL-6 on the area of the sensor functionalized for GFAP alongside the sensor response to GFAP at the same concentration were also compared. For both low and high concentrations of GFAP and IL-6, the specific response was significantly higher than the non-specific response. From this, it can be concluded that there is minimal cross-reactivity between GFAP and IL-6 that does not interfere with the specific signals of their respective markers.

### 3.7. Reproducibility Study

**Figure 7:**
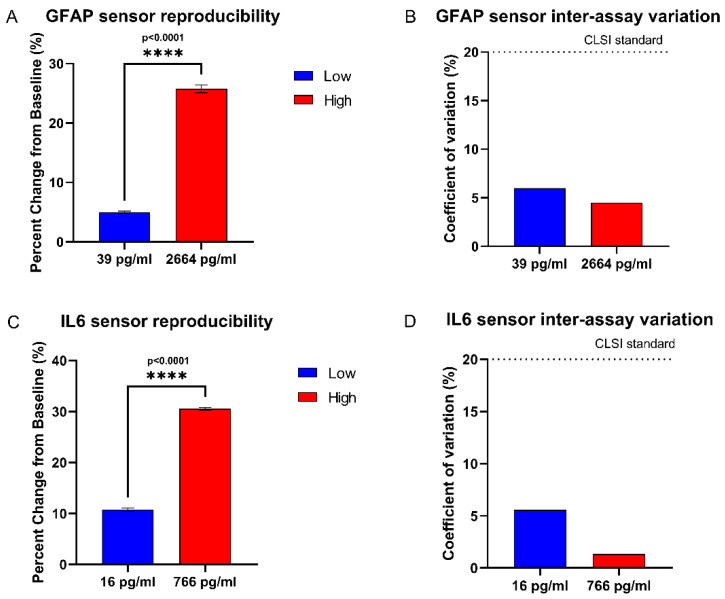
(**A**) Sensor response to triplicate measurements of GFAP on separate sensing units. (**B**) Coefficient of variation between triplicate measurements of GFAP on separate sensing units. (**C**) Sensor response to triplicate measurements of IL-6 on separate sensing units. (**D**) Coefficient of variation between triplicate measurements of IL-6 on separate sensing units.

The sensor was evaluated for reproducibility by taking triplicate measurements of the sensor response to synthetic sweat spiked with low and elevated concentrations of GFAP and IL-6 on separate sensing units. This was done to evaluate whether the variation between separate sensing units is significant, which would make the results of one sensing unit irreproducible. For the sensor results to be reproducible, the variation in sensor readings for the same concentration would need to be within the acceptable clinical threshold [40]. Figure 7A,B depicts the sensor response as a percentage change from baseline impedance. An unpaired t-test between the sensor response to low and high concentrations of both markers showed the sensor response to be highly significant between low and high concentrations, thus showing that the sensor can distinguish between high and low concentrations of both markers. Figure 7C,D depicts the coefficient of variation across the repeat measurements of GFAP and IL-6, which are below 20%; the acceptable clinical standard for intra assay variation, according to the Clinical Laboratory Standards Institute [40]. From this, it can be inferred that variation between sensor measurements on separate sensing units is within the acceptable range for both low and elevated concentrations of both markers, and that variation between sensing units does not significantly affect measured concentration.

### 3.8. Proof of Feasibility Study in Interpreting Spiked TBI Conditions in Human Sweat

**Figure 8:**
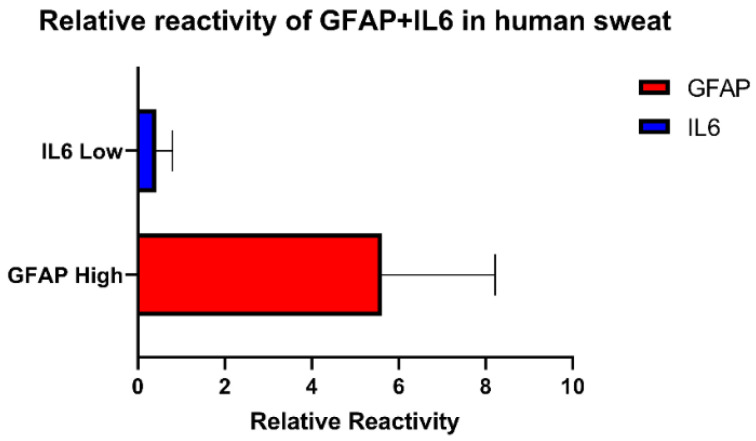
Relative reactivity of GFAP and IL-6 for a simulated TBI in human sweat.

In real life conditions, immediately after the onset of injury, IL-6 possesses a slower response time than GFAP due to its release being an immune response [12]. Whereas GFAP, which is released immediately upon rupturing of the blood–brain barrier due to injury, experiences a dramatic spike within 2–3 h of injury [7]. We hypothesize that the post-injury temporal profiles of both markers in sweat would follow the same trend as seen in serum post-injury. In order to simulate the sweat of a TBI patient 2–3 h post-injury, human sweat samples were spiked together with a high concentration of GFAP and a low concentration of IL-6 consistent with the estimated physiological ranges of both markers in sweat for injured individuals. The concentrations used were 2000 pg/mL of GFAP (high concentration) and 0.78 pg/mL of IL-6 (low concentration), which are within the upper and lower limits of their estimated ranges in sweat. The sensor response was represented as relative reactivity, which was calculated as the ratio of the measured concentration to the actual concentration of the sample. Figure 8 shows that the relative reactivity of a high concentration of GFAP as measured by the sensor is higher than the relative reactivity of a low concentration of IL-6 as measured by the sensor in the same sample of human sweat, which simulates the relative levels of GFAP and IL-6 that would we hypothesize would be present in a TBI patient 2–3 h post-injury.

## 4. Conclusions

In this work, we have demonstrated a flexible, sweat-based sensor, SWEATSENSER, for measuring prognostic TBI markers relevant to monitoring of a TBI post-injury. The calibrated ranges are consistent with the physiological expression ranges of GFAP and IL-6. Calibration studies carried out using EIS were validated with similar results using chronoamperometry. Spike and recovery studies showed a significant response of the recovered concentrations within the concentration range used to calibrate the sensor. Reproducibility and cross-reactivity studies established the accuracy and specificity of the sensor. The sensor demonstrated the ability to detect the relative reactivities of GFAP and IL-6 in simulated profiles of mild and moderate TBIs in human sweat. Future work in this area would require clinical studies of TBI patients to establish the presence of GFAP in human sweat post-injury and generate a post-injury profile for both markers.

## Figures and Tables

**Table 1:** Summary of physiological ranges of GFAP and IL-6 in sweat and serum.

Biofluid	Levels (pg/mL)			
	GFAP		IL-6	
	**No Injury**	**Injured**	**No Injury**	**Injured**
**Serum**	0–61 [19]	8–2000 [7]	0–43.5 [20]	0.8–500 [21]
**Sweat**	0–15 *	10–540 *	0.01–15 [22]	6–162 [15]

* Estimated range in sweat.

## Data Availability

Not applicable.
